# Risk of rheumatoid arthritis diagnosis in statin users in a large nationwide US study

**DOI:** 10.1186/s13075-021-02617-5

**Published:** 2021-09-18

**Authors:** Madeline N. Peterson, Hayley J. Dykhoff, Cynthia S. Crowson, John M. Davis, Lindsey R. Sangaralingham, Elena Myasoedova

**Affiliations:** 1 Division of Rheumatology, Mayo Clinic, 200 First Street SW, Rochester, MN 55905 USA; 2 Robert D. and Patricia E. Kern Center for the Science of Health Care Delivery, Mayo Clinic, 200 First Street SW, Rochester, MN 55905 USA; 3 Department of Quantitative Health Sciences, Mayo Clinic, 200 First Street SW, Rochester, MN 55905 USA

**Keywords:** Rheumatoid arthritis, Lipid-lowering medications, Risk factors

## Abstract

**Objective:**

To evaluate the association between statin use and the risk of developing rheumatoid arthritis (RA) in a large, US case-control study.

**Methods:**

Using the OptumLabs Data Warehouse, RA cases were identified as patients aged ≥18 years with ≥2 RA diagnoses between January 1, 2010 and June 30, 2019 and ≥1 prescription fills for methotrexate within 1 year of the first RA diagnosis. The first RA diagnosis was the index date. Cases were matched 1:1 to controls on age, sex, region, year of index date, and length of baseline coverage. Statin users were defined by having ≥2 statin prescription fills at least 90 days pre-index. Patients identified as statin users were further classified by statin user status (current or former), statin use duration, and intensity of statin exposure. Odds ratios for RA risk with statin use were estimated using logistic regression.

**Results:**

16,363 RA cases and 16,363 matched controls were identified. Among RA cases, 5509 (33.7%) patients were statin users compared to 5164 (31.6%) of the controls. Statin users had a slightly increased risk of RA compared to non-users (OR 1.12, 95% CI 1.06–1.18), and former statin users had an increased RA risk compared to current users (OR 1.21, 95% CI 1.13–1.28). However, risk was eliminated following adjustment for hyperlipidemia. The risk estimates for statin use duration and intensity did not reach significance.

**Conclusion:**

This study demonstrates no significant increase in the risk of developing RA for statin users compared to non-users after adjustment for hyperlipidemia in addition to other relevant confounders. However, more information from prospective studies would be necessary to further understand this relationship.

**Supplementary Information:**

The online version contains supplementary material available at 10.1186/s13075-021-02617-5.

## Background

Statins, or 3-hydroxy-3-methylglutaryl coenzyme A (HMG-CoA) reductase inhibitors, are among the most widely prescribed drugs in the US [1] and are proven to reduce the risk of cardiovascular morbidity and mortality by inhibiting cholesterol synthesis [2–6]. In addition, there is growing data on the pleiotropic effects of statin use. Statins have been shown to modify a range of non-lipid-related cell signaling pathways, including those involved in eliciting inflammatory responses [7, 8].

The anti-inflammatory and immunomodulatory effects of statins have been observed in randomized controlled trials and observational studies in the general population [9] and in patients with chronic inflammatory diseases, such as rheumatoid arthritis (RA) [10–13]. In RA patients, statins have been shown to reduce C-reactive protein levels, joint inflammation, and overall disease activity [12, 14–16].

In contrast, results of a systematic review compiling reports from long-term statin users found that statins may induce autoimmunity and predispose patients to the development of rheumatic conditions, such as systemic lupus erythematosus, dermatomyositis, and polymyositis [17]. Literature regarding the role of statins in the development of RA is conflicting with studies reporting a harmful [18, 19], protective [20–22], or neutral effect [23–25] on incident RA. A recent meta-analysis summarizing findings from observational studies in mainly European populations showed no difference in the risk of RA in statin users versus non-users [26]. Large studies examining the association between statin use and RA risk in the US are lacking.

This nation-wide case-control study aimed to evaluate the effects of statins on the development of RA and intended to specifically assess the influence of statin use duration and intensity, as well as the potential confounding of comorbidities, particularly hyperlipidemia.

## Methods

### Data source

This study used the OptumLabs Data Warehouse, a large administrative database containing de-identified health information for commercially insured and Medicare Advantage beneficiaries. This database represents a diverse mixture of ages, races/ethnicities, and geographical regions across the US and contains longitudinal information on enrollees, such as medical and pharmacy claims, laboratory results, and enrollment records [27]. Institutional Review Board approval was not required because the data have been de-identified.

### Case definition and selection

**Fig. 1:**
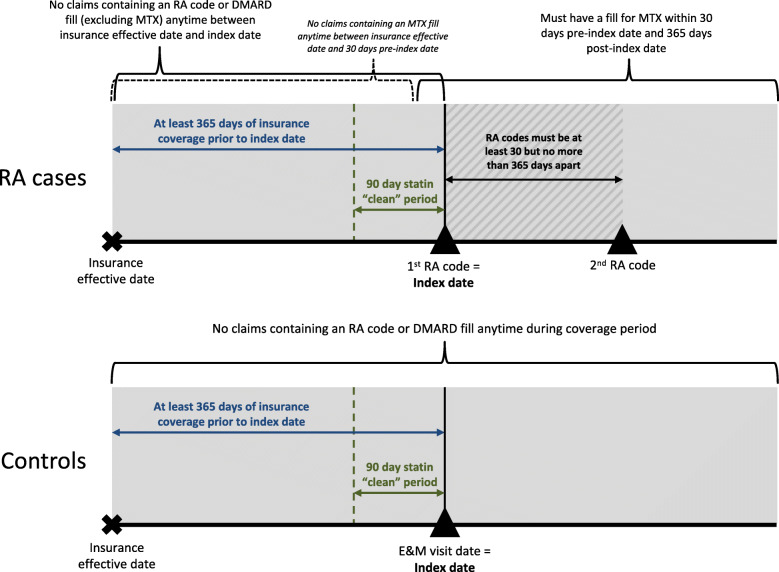
Study design for RA case and control selection. RA rheumatoid arthritis, DMARD disease-modifying antirheumatic drug, MTX methotrexate, E&M evaluation & management

Cases were defined as individuals aged ≥18 years who had two or more claims containing an RA diagnostic code [*International Classification of Disease (ICD)-9*: 714.0, 714.1, 714.2; *ICD-10*: M05.xxx, M06.0xx, M06.8x, M06.9] between January 1, 2010 and June 30, 2019 with diagnoses being at least 30 days but no more than 365 days apart and one or more prescription fills for methotrexate (MTX) within 30 days before and 365 days after the first RA diagnosis. RA cases were required to have at least 365 days of medical and pharmacy insurance coverage prior to the first RA diagnosis; patients without this baseline period of coverage were considered prevalent RA patients and were excluded. Patients were excluded if they had an RA diagnostic code and/or a prescription fill for a disease-modifying antirheumatic drug (DMARD) any time during coverage prior to the first RA diagnosis and/or a prescription fill for MTX any time prior to 30 days before the first RA diagnosis. The date of the first RA diagnostic code was used as the index date (Fig. 1).

### Control definition and matching

Controls were individuals aged ≥18 years who had one or more claims for an evaluation and management visit during the study period and at least 365 days of baseline insurance coverage prior to the claim date. Of the claims that met the insurance criteria, one was randomly selected as the index date for each patient. Patients with an RA diagnostic code and/or DMARD prescription fill (including MTX) any time during their insurance coverage were excluded. Controls were matched 1:1 with RA cases on exact age, sex, geographical region, calendar year of index date, and length of baseline insurance coverage (within 30 days).

### Statin use assessment

Statin users and non-users were identified using prescription fill data. Statin users were patients with two or more statin fills between the start of insurance coverage and 90 days pre-index date. Patients with one or zero fills during this period and those who started statins within 90 days of the index date were considered non-users. For every statin prescription fill, the count day supply included in the prescription fill was defined as the number of statin days (NSD). Statin users with remaining statin supply based on the prescription fill and NSD at the time of index were considered current users. Statin users without available statin supply at the time of index were considered former users. In addition, statin users with a statin fill during the first 120 days of insurance coverage were considered prevalent users; users who did not have a first statin fill until after this period were considered incident users.

The total duration of statin use was determined by linking the patients’ statin prescription fill dates and summing the NSD periods. If the next prescription fill date was within 30 days of the NSD, the patient was considered a continuous user, and the periods between prescription fills were summed into a single duration. If the patient did not have a subsequent statin fill within 30 days of the NSD, the last day of the patient’s statin supply was recorded as the statin stop date. Patients who stopped and later resumed statin therapy, either once or multiple times, may have several start and stop dates. For these patients, the periods between each set of start and stop dates were summed to calculate the total duration of statin use. Duration of statin use was divided into three categories: <1 year, ≥1 year, or unknown. Because prevalent users had a statin fill within the first 120 days of coverage, the true statin start date was unknown, and it was not possible to determine the total duration of statin use for these patients. Prevalent users who used statins for less than 1 year during coverage were placed into the unknown duration group. However, prevalent users who used statins for 1 year or more during coverage could still be accurately placed into the ≥1 year duration group.

The intensity of statin exposure was also evaluated. Using existing guidelines [28], statin intensity was divided into three categories according to the typical low-density lipoprotein cholesterol (LDL-C) reduction level: ≥50% reduction was classified as high intensity, 30–49% as medium, and <30% as low. A detailed table of intensity classifications for various medications and dosages is available online (Table S1). Statin intensity for each patient was defined according to the highest intensity statin ever filled by the patient.

### Potential confounders

Diagnostic codes were used to compute the Charlson and Elixhauser Comorbidity Indices [29, 30], which are well-established methods for quantifying the burden of comorbidity. Rheumatic disease was omitted from both indices. Hyperlipidemia was also defined using diagnostic codes. All baseline comorbidities were identified via ICD-9/10 diagnostic codes during a screening period from 365 days pre-index date through 1 day pre-index date. Obesity and smoking were also defined using diagnostic codes any time between the insurance effective date and the index date, as data on body mass index (BMI) and patient-reported smoking status were not available for the majority of patients. The diagnostic code set to identify smoking status was developed previously [31]. For the subset of patients with available laboratory data, the closest LDL-C level measured prior to the index date was used in the analysis.

### Statistical analysis

Descriptive statistics were used to summarize the data. Logistic regression models were used to estimate odds ratios (OR) and 95% confidence intervals (CI) for the effect of statin use on the development of RA. Outcomes were adjusted for age, sex, race/ethnicity, calendar year of index date, and geographical region. In addition, two models included comorbidity adjustments using the Charlson and Elixhauser Comorbidity Indices.

Numerous sensitivity analyses were performed with regard to outcome definition, inclusion criteria, intensity assessment, and potential confounders. Two sensitivity analyses were performed for the RA definition: one analysis specified that the RA diagnosis must come from a rheumatologist, and a second analysis loosened the original definition by allowing RA patients to use any DMARD in the year after diagnosis, instead of strictly MTX. To assess the effect of age on RA risk, a subgroup analysis was performed observing the risk in patients aged ≥40 years. To investigate the potential confounding of hyperlipidemia, two analyses were performed. First, risk estimates were individually adjusted for hyperlipidemia via diagnostic codes (ICD-9 272.0–272.4, ICD-10 E78.0x–E78.5). Second, an analysis was performed using a subset of the original population who had laboratory data for lipid levels. Risk estimates for this subgroup were adjusted for LDL-C levels instead of hyperlipidemia diagnostic codes. To assess any residual confounding of comorbidities, a sensitivity analysis adjusted for the following ten conditions as independent variables: obesity, myocardial infarction, cerebrovascular disease, congestive heart failure, peripheral vascular disease, diabetes, hypothyroidism, liver disease, metastatic cancer, and renal failure. The influence of smoking was also investigated. Lastly, an analysis was performed using the intensity of the most recently filled statin as the intensity variable. Analyses were performed using SAS version 9.4 (SAS Institute, Cary, NC, USA).

## Results

**Fig. 2:**
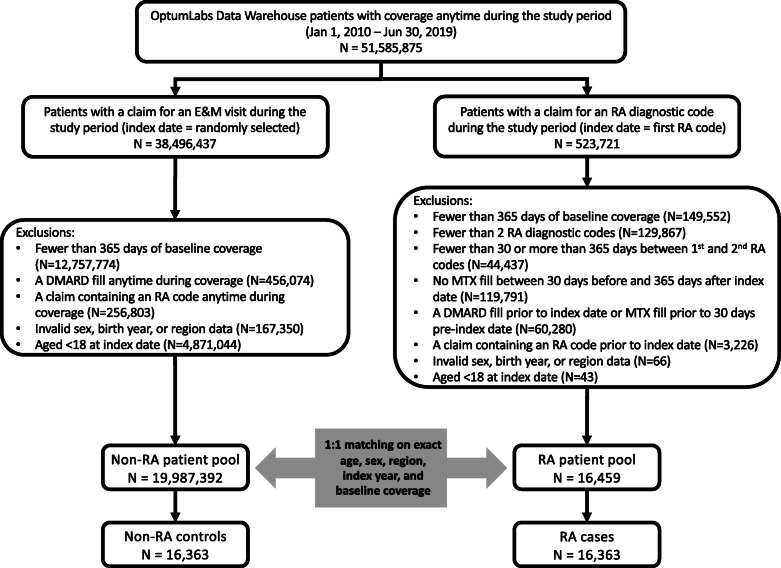
Study population flow diagram. E&M evaluation and management, RA rheumatoid arthritis, MTX methotrexate, DMARD disease-modifying antirheumatic drug

Using the OptumLabs Data Warehouse, 51,585,875 patients with Medicare Advantage or commercial medical and pharmacy enrollment between January 1, 2010 and June 30, 2019 were identified. Of these, 523,721 had a claim containing an RA code during the study period. Excluding patients who did not meet the inclusion criteria yielded 16,459 eligible RA patients. There were 19,987,392 eligible control patients. After 1:1 matching, 16,363 RA cases and 16,363 controls were included in this study (Fig. 2).

Cases and controls had equivalent or similar baseline characteristics and comparable distributions of comorbidities (Table 1). Both populations consisted of predominantly white females (70.7% female, RA cases 73.6% white, non-RA controls 74.2% white). Among RA cases, 5509 (33.7%) were found to be statin users versus 5164 (31.6%) of the controls (Table 2). The distribution of current and former users among cases and controls was similar; there were slightly more former users among RA cases and slightly more current users among controls. The majority of statin users, with and without RA, had at least one full year of statin use at the time of index. In addition, the highest statin dosage ever filled for most statin users was a medium intensity statin.

**Table 1:** Baseline characteristics of rheumatoid arthritis cases and controls

	Cases (***n*** = 16,363)	Controls (***n*** = 16,363)
**Age (years), mean (SD)**	58.2 (14.1)	58.2 (14.1)
**Sex,*n*(%)**		
Female	11565 (70.7)	11565 (70.7)
Male	4798 (29.3)	4798 (29.3)
**Race/ethnicity,*n*(%)**		
Asian	437 (2.7)	640 (3.9)
Black	1572 (9.6)	1542 (9.4)
Hispanic	1822 (11.1)	1448 (8.8)
White	12039 (73.6)	12147 (74.2)
Other/unknown	493 (3.0)	586 (3.6)
**Region,*n*(%)**		
Midwest	4185 (25.6)	4185 (25.6)
Northeast	1800 (11.0)	1800 (11.0)
South	8185 (50.0)	8185 (50.0)
West	2193 (13.4)	2193 (13.4)
**Baseline insurance coverage (years), mean (SD)**	4.1 (3.0)	4.1 (3.0)
**Disease history,*n*(%)**		
Hyperlipidemia	7848 (48.0)	6935 (42.4)
Myocardial infarction	378 (2.3)	423 (2.6)
Cerebrovascular disease	1086 (6.6)	1038 (6.3)
Congestive heart failure	793 (4.8)	887 (5.4)
Peripheral vascular disorders	1326 (8.1)	1177 (7.2)
Diabetes	3331 (20.4)	3092 (18.9)
Hypothyroidism	3481 (21.3)	2687 (16.4)
Liver disease	783 (4.8)	676 (4.1)
Metastatic cancer	74 (0.5)	240 (1.5)
Obesity	2466 (15.1)	1917 (11.7)
Renal failure	862 (5.3)	993 (6.1)
**Charlson index score, mean (SD)**	2.2 (1.9)	2.3 (2.2)
**Elixhauser index score, mean (SD)**	2.4 (2.3)	2.2 (2.5)

**Table 2:** Risk of rheumatoid arthritis in statin users compared to non-users

Exposure	Cases (***n*** = 16,363)	Controls (***n*** = 16,363)	OR^**1**^ (95% CI)	OR^**2**^ (95% CI)	OR^**3**^ (95% CI)	OR^**4**^ (95% CI)
**Statin user status**						
Non-user	10854	11199	1.00	1.00	1.00	1.00
Statin user	5509	5164	1.12 (1.06, 1.18)	1.14 (1.08, 1.20)	1.07 (1.01, 1.13)	0.95 (0.90, 1.01)
Current user	2629 (47.7)	2680 (51.9)	1.03 (0.97, 1.10)	1.05 (0.98, 1.12)	0.98 (0.92, 1.05)	0.87 (0.81, 0.93)
Former user	2880 (52.3)	2484 (48.1)	1.21 (1.13, 1.28)	1.23 (1.15, 1.31)	1.15 (1.08, 1.23)	1.03 (0.96, 1.10)
**Statin use duration**						
<1 year statin use	996 (18.1)	856 (16.6)	1.20 (1.09, 1.33)	1.22 (1.11, 1.34)	1.16 (1.05, 1.27)	1.04 (0.94, 1.15)
≥1 year statin use	3917 (71.1)	3795 (73.5)	1.08 (1.02, 1.15)	1.10 (1.04, 1.17)	1.04 (0.98, 1.10)	0.91 (0.86, 0.97)
Unknown	596 (10.8)	513 (9.9)	1.20 (1.07, 1.36)	1.23 (1.09, 1.39)	1.14 (1.01, 1.29)	1.02 (0.90, 1.16)
**Statin use intensity**						
Low	519 (9.4)	505 (9.8)	1.08 (0.95, 1.23)	1.10 (0.97, 1.25)	1.04 (0.91, 1.18)	0.93 (0.82, 1.06)
Medium	3486 (63.3)	3290 (63.7)	1.11 (1.05, 1.18)	1.12 (1.06, 1.19)	1.07 (1.01, 1.13)	0.95 (0.89, 1.01)
High	1504 (27.3)	1369 (26.5)	1.15 (1.06, 1.25)	1.18 (1.09, 1.28)	1.08 (1.00, 1.18)	0.96 (0.88, 1.05)

^1^Model 1: Adjusted for age, sex, race/ethnicity, calendar year of index date, and geographical region

^2^Model 2: Model 1 + adjustment for Charlson Comorbidity Index

^3^Model 3: Model 1 + adjustment for Elixhauser Comorbidity Index

^4^Model 4: Model 3 + adjustment for hyperlipidemia

Statin use was associated with a modest increase in the risk of RA (OR 1.12, 95% CI 1.06–1.18 [adjusted for age, sex, race/ethnicity, index year, and region]). This difference persisted following additional adjustment for the Charlson (OR 1.14, 95% CI 1.08–1.20) and Elixhauser (OR 1.07, 95% CI 1.01–1.13) Comorbidity Indices. However, this increased risk was eliminated following additional adjustment for hyperlipidemia (OR 0.95, 95% CI 0.90–1.01). Compared to those who did not use statins, former users had a greater risk of developing RA (OR 1.21, 95% CI 1.13–1.28), whereas current statin users had no increase in risk (OR 1.03, 95% CI 0.97–1.10). Following adjustment for hyperlipidemia, former users had no increased RA risk (OR 1.03, 95% 0.96–1.10) and current users had a slightly decreased risk (OR 0.87, 95% CI 0.81–0.93). Patients who used statins for <1 year had a slightly increased risk compared to those who used statins for ≥1 year, but this difference did not reach statistical significance in any of the models. A slight dose-response relationship between statin intensity and RA was observed. However, this trend did not reach significance. Results were similar in the analysis for the intensity of the most recently filled statin (Table S2).

In the additional sensitivity analysis for hyperlipidemia, which included only individuals who had laboratory data for LDL-C, 6948 RA cases and 6948 matched controls were identified. In both cases and controls, 2862 (41.2%) individuals were found to be statin users. In this subgroup assessment, the risk of RA, prior to additional adjustment, was not elevated (OR 1.00, 95% CI 0.93–1.08). When risk estimates were individually adjusted for LDL-C level, they remained unchanged. Sensitivity analyses for RA definition, age, additional comorbidities, and smoking produced results consistent with the initial findings (Table S2).

## Discussion

To the authors’ knowledge, this is the first large case-control study to evaluate the relationship between statin use and RA risk in the US. Findings of this study demonstrate that statin use is associated with a modest increase in the risk of developing RA, but following adjustment for hyperlipidemia, this risk is diminished. Former statin users had an increased risk of RA versus current statin users. Patients who used statins for <1 year were found to have a slightly increased risk of RA versus those who used statins for ≥1 year. A slight dose-response relationship was observed for statin intensity and RA risk. However, the trends for statin use duration and intensity did not reach statistical significance, and no subgroup had a meaningfully increased risk following hyperlipidemia adjustment.

The reduction in risk seen with hyperlipidemia adjustment warrants further discussion. Prior literature suggests that hyperlipidemia may serve as an independent risk factor for RA [21], and the results of this study appear to support the notion that an increased RA risk may be due to hyperlipidemia and not statin use. However, it is important to consider that many patients receive a hyperlipidemia diagnosis during the initiation of statin treatment, and therefore, any attenuation in risk resulting from hyperlipidemia adjustment may not reflect a true association. With the exception of one prior study [19], no comparative study reported an analysis that specifically assessed the influence of hyperlipidemia/cholesterol values as an independent variable in adjustments. Therefore, it is unknown how hyperlipidemia impacted risk estimates in prior studies and if any consequential change in risk reflected a true association.

If the reduction in risk following hyperlipidemia adjustment is artifactual, the underlying mechanism for how statins may facilitate RA development is unknown. Some evidence suggests that statins may disrupt immune homeostasis by directly affecting T cells and inducing a shift from Th1 to Th2 immune responses [32, 33]. This may lead to B cell hyperactivity and trigger the production of autoantibodies [34, 35]. In addition, the burden of infections over time, compounded by a reduced Th1 response, may foster breakdown of self-tolerance, as infectious agents cannot be cleared as effectively as they would be under normal conditions [36]. Another possibility is that statins, being pro-apoptotic agents, may be capable of activating or exacerbating cellular apoptosis [37–41]. The release of endoplasmic and endonuclear antigens into circulation during cell death may induce the production of pathogenic autoantibodies [42, 43]. Cardiovascular risk factors, such as hyperlipidemia, may also play a role, as data has shown that patients who later develop RA tend to have significantly more atherogenic lipid profiles [44]. Therefore, statin use may be a proxy for hyperlipidemia, and thus, the unfavorable lipid conditions may be responsible for the association with increased RA risk, potentially through the chain of proinflammatory and immunogenic reactions [45–47]. These mechanisms for loss of tolerance would likely not induce autoimmunity on their own, but they may result in an earlier diagnosis or influence the progression of a condition, such as RA, in those who are already prone to the development of an autoimmune disease [18].

Of the studies that have previously investigated this topic, two found statins to have a harmful effect, three demonstrated a protective effect, and three observed no clear impact on the risk of incident RA. A retrospective cohort study of 511,620 statin users, which observed an increased risk of RA in statin users compared to non-users, also observed a greater RA risk for recent users compared to current users [19]; this aligns with the observation of an increased RA risk in former versus current users in the present study. With the exception of this one similarity, the present study does not seem to clearly support or refute the findings from any prior study that has investigated this topic, as the results have been largely heterogeneous overall. Therefore, it seems necessary to further discuss key methodological differences between the present and prior studies that could account for the conflicting data.

First, lack of non-statin reference groups: Two prior studies, showing a protective effect of statins against RA, compared statin users depending on persistence with treatment [20] and treatment intensity [22], precluding direct comparison with the current study.

Second, variability in definitions of outcome and exposure: Compared to prior studies, the present study used a more specific and exclusive RA definition that was selected in order to avoid misclassifying patients with conditions that present similarly to RA, as this may have been an issue in other studies. Sensitivity analyses with regard to the RA definition produced similar results. The definition of statin exposure also varied among studies. While the current study required at least two statin prescription fills for statin users, several prior studies required only one statin fill or did not define a minimum exposure requirement [19, 20, 22–25]. Some of these studies may have included users with near negligible periods of statin exposure, which may explain the inconsistencies in the findings. When the statin user requirement was changed to only one prescription fill, the results remained unchanged. However, in this study, statin users were identified by the number of statin fills, while other studies had to rely on prescription data without information on whether the statin was actually filled. Therefore, it is possible that a similar change to the statin user definition may have a meaningful impact on risk estimates in other studies. Lastly, there was variation in the considerations for potential lag-times. Assuming that statin exposure is unlikely to influence the risk of RA immediately after initiating treatment, RA cases diagnosed within 90 days of starting statins were excluded. Previous studies that did not account for this delay may have included patients whose RA symptoms predated the initiation of statin therapy. There is also an assumed delay between symptom onset and RA diagnosis. Chan et al. [48] estimated a median delay from RA onset to diagnosis of approximately 36 weeks; thus, many patients may have developed RA months or years prior to the recorded event date. However, several previous studies have considered the impacts of this delay, and the results remained largely unchanged in all studies [18, 19, 21, 22].

Third, consideration for potential confounders: Similar to four previous studies [18–20, 22], the current study excluded RA patients with DMARD use before the index date and non-RA patients with any DMARD use. Studies not excluding for DMARD use may have resulted in inaccurate classifications of RA and non-RA patients. This study considered a more comprehensive list of pre-existing conditions as potential confounders through the use of two comorbidity indices. Adjustments for the Charlson and Elixhauser Comorbidity Indices did not significantly alter the results. One other study accounted for a comorbidity index [24], but the reported comorbidity scores were much lower than in the present study. This was likely due to the younger age of patients in that study conducted within a military health care system and may explain the result discrepancies with the present study. When ten additional comorbidities were added to adjustments as independent variables, the risk remained unchanged. Consideration for the influence of hyperlipidemia, as previously mentioned, was another important factor that may explain the different results.

Simvastatin 10mg has been available over-the-counter since 2004 in the UK. Four studies were conducted in UK populations with study periods during this time [19, 22, 23, 25]. The current study was performed in the US, where statins are available via prescription only. Over-the-counter statin use may have led to an underestimation of statin exposure in both user and non-user populations. It is unknown how this may have impacted the findings in those studies.

Fourth, biases: Berkson’s bias may have influenced the results, as joint pains may have prompted a thorough work-up that resulted in statin initiation prior to a subsequent diagnosis of RA. Ascertainment bias may have also influenced the results. As noted by de Jong and colleagues [19], some patients who recently begin statin therapy may experience muscle-related symptoms [49], which may lead to a greater number of clinical visits, including rheumatology referrals. Thus, statin users may have been more likely to be diagnosed with RA than non-users.

Strengths of this study include its large and representative sample size with >16,000 RA patients, more than any prior study on this topic. The strong comparability due to exact matching of cases and controls likely reduced measured and unmeasured confounding. A large range of sensitivity analyses were performed, producing largely consistent results, which suggests minimal impact of residual confounders. Lastly, this study was specifically designed to assess RA as an outcome of statin use by comparing statin users to non-users. Many of the prior studies examined additional outcomes or did not utilize a non-user reference group.

There are several potential limitations of this study. First, this was a retrospective, observational study, and the use of statins was not randomized. In addition, statin use was determined via prescription fill data. However, as previously mentioned, many administrative data sources rely on prescription data without information on whether the prescription was actually filled, so others may have overestimated statin use even more than the current study. Case-control studies are limited in that they cannot be used to calculate incidence and are not suitable to imply causation. However, considering the low prevalence of RA among the general population, a case-control study seemed to have greater power to assess this association compared to alternatives, such as a retrospective cohort study. Limited information was available on rheumatoid factor or anti-cyclic citrullinated peptide antibodies and on lifestyle factors (e.g., diet, physical activity), which may be important risk factors for RA [50, 51]. Adjustments for smoking and obesity did not have an impact on risk estimates. However, smoking, obesity, and comorbidities were determined via diagnostic codes and could not be verified with clinical data, such as BMI, blood pressure, glucose levels, or inflammatory markers. Finally, duration and intensity assessments were limited by the large number of prevalent statin users in this study. The duration of statin use was unknown for some of these patients, so the actual number of individuals in both duration groups should be higher. Although the sensitivity analysis for intensity produced consistent results, it was not possible to accurately perform a cumulative exposure assessment to examine the impacts of differential statin intensity use over time.

## Conclusion

In summary, the results of this nation-wide study suggest there is no significant increase in the risk of RA occurrence in statin users, adjusting for hyperlipidemia in addition to other relevant confounders. The current body of conflicting data on this topic demonstrates the challenge of performing a retrospective analysis on the relationship between a prescription drug and an unrelated medical disease. This endeavor becomes increasingly difficult in the case of RA due to its minimal prevalence in the general population, shaded symptomology, and multifactorial etiology. Taken together with the heterogeneous results of other reports, this study does not indicate or support any change to the current clinical recommendations for prescribing statins.

## Supplementary Information

**Additional file 1: Supplementary Table 1**. Intensity classification for various statin medications and dosages

**Additional file 2: Supplementary Table 2**. Sensitivity analyses to assess the stability of findings

## Data Availability

The data used and analyzed in the current study are available from OptumLabs through the OptumLabs Data Warehouse (OLDW). Restrictions apply to the availability of these data, which were used under license for the current study and therefore are not publicly available. However, the data are available from the authors upon reasonable request and with permission from OptumLabs.

## Abbreviations

HMG-CoA: 3-Hydroxy-3-methylglutaryl coenzyme A
RA: Rheumatoid arthritis
ICD: International classification of disease
MTX: Methotrexate
DMARD: Disease-modifying antirheumatic drug
NSD: Number of statin days
LDL-C: Low-density lipoprotein cholesterol
BMI: Body mass index
OR: Odds ratio
CI: Confidence interval
