# Supplementary material for: Risk of rheumatoid arthritis diagnosis in statin users in a large nationwide US study
Source: Arthritis Res Ther. 2021 Sep 18;23:244. doi: 10.1186/s13075-021-02617-5 (PMC8449497; doi:10.1186/s13075-021-02617-5)
Supplement: Supplementary file 1 — Additional file 1: Supplementary Table 1. Intensity classification for various statin medications and dosages [file 13075_2021_2617_MOESM1_ESM.docx]

**Supplementary Table 1.** Intensity classification for various statin medications and dosages

|  | **High intensity** | **Medium intensity** | **Low intensity** |
| --- | --- | --- | --- |
| **LDL-C reduction*** | ≥50% | 30-49% | <30% |
| **Statins** | Atorvastatin 40-80mg | Atorvastatin 10-20mg | Simvastatin 10mg |
|  | Rosuvastatin 20-40mg | Rosuvastatin 5-10mg | Pravastatin 10-20mg |
|  |  | Simvastatin 20-40mg | Lovastatin 20mg |
|  |  | Pravastatin 40-80mg | Fluvastatin 20-40m |
|  |  | Lovastatin 40-80mg |  |
|  |  | Fluvastatin 40mg BID |  |
|  |  | Fluvastatin XL 80mg |  |
|  |  | Pitavastatin 1-4mg |  |

*Average LDL-C reduction that is expected with daily dose
