# Supplementary material for: Risk of rheumatoid arthritis diagnosis in statin users in a large nationwide US study
Source: Arthritis Res Ther. 2021 Sep 18;23:244. doi: 10.1186/s13075-021-02617-5 (PMC8449497; doi:10.1186/s13075-021-02617-5)
Supplement: Supplementary file 2 — Additional file 2: Supplementary Table 2. Sensitivity analyses to assess the stability of findings [file 13075_2021_2617_MOESM2_ESM.docx]

**Supplementary Table 2.** Sensitivity analyses to assess the stability of findings

| **Sensitivity analysis** | **OR^1^**  **(95% CI)** | **OR^2^**  **(95% CI)** | **OR^3^**  **(95% CI)** | **OR^4^**  **(95% CI)** | **OR***  **(95% CI)** |
| --- | --- | --- | --- | --- | --- |
| 1. Restrict to patients 40 years and older | 1.11 (1.06, 1.17) | 1.13 (1.07, 1.19) | 1.07 (1.02, 1.13) | 0.96 (0.91, 1.02) |  |
| 2. Change intensity variable to the intensity of the most recently filled statin | | | | |  |
| Low intensity | 1.09 (0.97, 1.22) | 1.11 (0.99, 1.24) | 1.04 (0.93, 1,16) | 0.93 (0.83, 1.04) |  |
| Medium intensity | 1.11 (1.05, 1.18) | 1.13 (1.07, 1.20) | 1.07 (1.01, 1.13) | 0.95 (0.89, 1.01) |  |
| High intensity | 1.15 (1.05, 1.26) | 1.18 (1.08, 1.29) | 1.08 (0.99, 1.18) | 0.96 (0.87, 1.05) |  |
| 3. Change statin user definition to patients with one or more statin fills | 1.14 (1.09, 1.20) | 1.16 (1.10, 1.22) | 1.09 (1.04, 1.15) | 0.97 (0.92, 1.03) |  |
| Current user | 1.04 (0.98, 1.11) | 1.06 (0.99, 1.13) | 0.99 (0.93, 1.06) | 0.87 (0.81, 0.94) |  |
| Former user | 1.23 (1.16, 1.31) | 1.25 (1.18, 1.33) | 1.18 (1.11, 1.25) | 1.05 (0.99, 1.13) |  |
| 4. *Includes additional comorbidities† as independent variables in Model 4 |  |  |  |  | 0.97 (0.91, 1.03) |
| Current user |  |  |  |  | 0.88 (0.82, 0.95) |
| Former user |  |  |  |  | 1.05 (0.98, 1.12) |
| 5. *Includes smoking as an independent variable in Model 4 |  |  |  |  | 0.94 (0.89, 0.99) |
| Current user |  |  |  |  | 0.86 (0.80, 0.92) |
| Former user |  |  |  |  | 1.01 (0.95, 1.09) |

1 Model 1: Adjusted for age, sex, race/ethnicity, calendar year of index date, and geographical region

2 Model 2: Model 1 + adjustment for Charlson Comorbidity Index

3 Model 3: Model 1 + adjustment for Elixhauser Comorbidity Index

4 Model 4: Model 3 + adjustment for hyperlipidemia

†obesity, myocardial infarction, cerebrovascular disease, congestive heart failure, peripheral vascular disease, diabetes, hypothyroidism, liver disease, metastatic cancer, and renal failure
